# Association Between Regional Cardiac Radiation Dose and Magnetic Resonance Imaging Myocardial Contractility Parameters: A Prospective Pilot Study

**DOI:** 10.3390/tomography12050070

**Published:** 2026-05-12

**Authors:** El-Sayed H. Ibrahim, Slade Klawikowski, Lindsay Puckett, Elizabeth Gore, Dayeong An, Jakub Bychowski, Antonio Sosa, Gerard Walls, Carmen Bergom

**Affiliations:** 1 Department of Radiology, Medical College of Wisconsin, Milwaukee, WI 53226, USA; asosa@mcw.edu; 2 Department of Radiation Oncology, Medical College of Wisconsin, Milwaukee, WI 53226, USA; sklawikowski@mcw.edu (S.K.); lpuckett@mcw.edu (L.P.); egore@mcw.edu (E.G.); cbergom@wustl.edu (C.B.); 3 Department of Radiology, Northwestern University, Chicago, IL 60611, USA; dan@mcw.edu; 4 Department of Cardiology, Medical University of Gdansk, 80-210 Gdansk, Poland; jakub.bychowski@gumed.edu.pl; 5 Patrick G Johnston Centre for Cancer Research, Queen’s University Belfast, Belfast BT9 7AE, UK; g.walls@qub.ac.uk; 6 Department of Radiation Oncology, Washington University School of Medicine, St. Louis, MO 63110, USA

**Keywords:** lung cancer, radiation therapy, radiation dose, magnetic resonance imaging, strain

## Abstract

Background/Objectives: Magnetic resonance imaging (MRI) provides a non-invasive means for a comprehensive assessment of the effect of radiation therapy (RT) on heart function. This study aims to determine RT induced cardiotoxicity in thoracic cancer patients using cardiac MRI. Methods: Cardiac MRI was performed at baseline and at six months post-treatment in patients undergoing standard-of-care RT for lung or esophageal cancers at a single institution. Parameters included regional myocardial strain in the longitudinal, circumferential, and radial directions as well as myocardium T1, T2, and extracellular-volume (ECV) maps. Cardiac segmental doses were extracted from the RT planning scans. The relationship between changes in segmental MRI parameters at six months and segmental heart RT dose were investigated. Results: Twelve patients underwent baseline MRI and four completed the follow-up MRI. Five of the segmental strain parameters showed notable changes between baseline and six-month follow-up. Increased doses in the heart base and apex were associated with moderate-to-large and mild deteriorations, respectively, in strain for all regions. Increased doses in the mid-ventricular regions were associated with improved strain in all regions. The segmental analysis revealed that myocardial regions nurtured by the left coronary artery are more negatively affected by radiation compared to those nurtured by the right coronary artery. Conclusions: Alterations in regional tissue and strain parameters on MRI vary according to local myocardial RT dose, suggesting there may be heterogeneity of radiation sensitivity for the heart substructures and regions. Changes in segmental strain parameters may reflect post-RT cardiac remodeling, but larger confirmatory studies are required.

## Simple Summary

The objective of this study is to determine the harm effects of radiation therapy on the heart in thoracic cancer patients using magnetic resonance imaging. The results showed alterations in regional heart function that varies according to local radiation dose to the heart muscle, suggesting that there may be heterogeneity of radiation sensitivity for the heart substructures and regions. Changes in regional heart contractility may reflect the heart’s response to radiation therapy, which may lead to better treatment planning in the future with fewer heart complications in cancer patients.

## 1. Introduction

Radiation therapy (RT), often combined with systemic therapy, plays an integral role in treating early and locally advanced lung and esophageal cancers. Although survival rates have increased due to the use of modern RT techniques and advances in treatments such as immunotherapy, radiation-induced cardiac toxicity is still a major concern in these patients [1]. Pre-clinical studies indicate that endothelial dysfunction, cardiomyocyte mitochondrial perturbations, inflammation, and fibrosis are drivers of cardiac toxicity [2]. Recent studies suggest that radiation-induced cardiac damage can manifest within two years of completing RT for patients with lung cancer [3,4,5,6], and that mortality correlates with mean heart dose [3], or with the percent of the heart receiving 5 Gy [7], 30 Gy, and 50 Gy [5]. However, other studies have found that cardiac substructure doses better correlate with outcomes [1,4,8]. Currently, it is unclear what cardiac substructure RT dose is safe, as a wide range of dose–volume parameters have been evaluated in the retrospective studies to date [1,9].

Currently, there are no validated clinical prediction tools for identifying patients at higher risk of cardiotoxicity from RT, and the clinical guidelines for baseline and follow-up cardiac care are not well-established [10]. In addition, current approaches for minimizing doses to organs at risk during RT planning use a one-model-fits-all approach, where a standard dose constraint is used to limit RT dose to the whole heart during treatment planning, regardless of baseline risk factor burden or the presumed diversity of relative radiation sensitivity among the cardiac substructures [4]. This strategy is particularly suboptimal in patients with lung and esophageal cancers, who receive high doses of thoracic radiation with large variability in dose distribution across different heart regions, and who typically have multiple cardiac risk factors and/or cardiac disease prior to treatment. Recent studies found that the dose received in the heart base area correlated with survival in patients with lung cancer [11,12]. Other studies found that the dose to the left anterior descending (LAD) coronary artery territory correlates with late cardiac toxicity [6,13,14]. These findings agree with recent preclinical studies showing regional changes in strain in areas given the same dose of radiation [15].

The current paradigm for cardiac function assessment and management of cardiovascular disease relies primarily upon the assessment of the left ventricle (LV) ejection fraction (EF). However, EF alone is limited in both its diagnostic and prognostic ability, as it may not reflect the underlying advancement of subclinical cardiovascular disease that could portend the development of treatment-induced heart dysfunction [16]. Therefore, identifying new biomarkers or imaging modalities capable of early detection and localization of subclinical cardiac dysfunction is essential for risk stratification and prompt intervention to avoid heart failure development. This may ultimately lead to better guidelines for RT planning to reduce cardiac damage and improve treatment outcomes. Magnetic resonance imaging (MRI) is considered the reference standard for the evaluation of cardiac function [17]. Cardiac MRI has been shown to be superior to echocardiography for identifying cardiotoxicity in cancer survivors [18]. Furthermore, advanced MRI techniques allow for tissue characterization, e.g., T1, T2, and extracellular volume (ECV) mapping, which have been associated with diffuse fibrosis and edema [19].

The value of regional cardiac dysfunction and reduced regional strain for predicting future cardiac events and heart failure is well established [20]. This has been shown in studies where MRI-generated strain detected abnormal myocardial function in cancer patients treated with chemotherapy, despite normal systolic function [21,22]. Furthermore, in a preclinical study, we recently identified regional myocardial strain by MRI as an early marker of RT-induced subclinical cardiac dysfunction despite a paradoxical increase in LVEF [15].

In this study, we conducted a prospective observational clinical study examining the effect of RT on regional myocardial tissue characteristics and strain in patients receiving definitive RT for non-small cell lung cancer and esophageal cancer. The results demonstrated that MRI-derived changes in regional myocardial strain parameters vary according to local myocardial RT dose, suggesting there may be heterogeneity of radiation sensitivity for the heart substructures and regions.

## 2. Materials and Methods

### 2.1. Study Design

This study was approved by our institutional review board (IRB), and written informed consent was obtained from all subjects. Twelve patients (9 males and 3 females; 9 non-small cell lung cancer (NSCLC) and 3 esophageal cancer) scheduled for RT were recruited to the study between July 2020 and June 2023. The patients underwent baseline (pre-treatment) and 6-month post-treatment follow-up MRI scans. Furthermore, a questionnaire about risk factors and comorbidities was collected from the patients at the first visit and combined with data extracted from medical records.

### 2.2. Treatment Planning and Dose Calculation

All patients underwent computed tomography (CT)-based treatment planning with position and immobilization per institutional standards. Patient setup reproducibility was achieved using appropriate clinical devices, generally Vac-Lok (vacuum-molded) bags. Daily treatment positioning was confirmed with cone beam CT scans. Planning CT scans were obtained with a uniform slice thickness of ≤3 mm (Siemens SOMATOM Drive, Erlangen, Germany). Intravenous contrast was encouraged for central lesions or those with nodal disease but left to the discretion of the treating physician. The radiation therapy plans were created in Monaco (Elekta AB, Stockholm, Sweden) using Monte Carlo-based dose calculation algorithms or Precision (Accuray, Madison, WI, USA) using convolution-superposition calculation algorithms. All lesions were assessed for respiratory motion using MIM version 7.2.9 (MIM Software Inc, Cleveland, OH, USA) to account for the tumor and nodal excursion with respiration, from a four-dimensional CT (4DCT) scan at the time of simulation. Respiratory motion management (RMM), including abdominal compression, AlignRT^®^-based breath hold (VisonRT, London, UK), and Synchrony motion management (Accuray, Madison, WI, USA), was considered for any lesion with motion > 1 cm.

**Figure 1:**
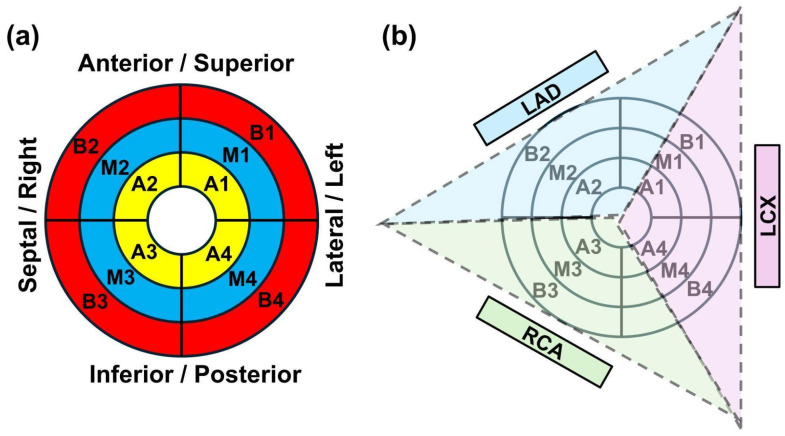
(**a**) Myocardium segmentation of the heart. The outer (red), middle (blue), and inner (yellow) circles represent basal, mid, and apical short-axis slices, divided into 4 segments each (B1 to B4, M1 to M4, and A1 to A4 to represent basal, mid, and apical segments, respectively). For each segment, circumferential, longitudinal, and apical strains were calculated. (**b**) LV myocardial territories nurtured by different coronary arteries, shown overlapped on the 12-segment model. Blue, purple, and green areas show myocardial territories nurtured by the left anterior descending (LAD) artery, left circumflex (LCX) artery, and right coronary artery (RCA), respectively.

If not originally included in the patient’s radiation therapy treatment plans, the aorta, pulmonary artery, left atrium, right atrium, left ventricle, right ventricle, heart, and pericardium contours were automatically contoured using MIM software, and then corrected and confirmed by both a radiation oncologist and medical physicist, as per methodology outlined in Duane et al. [23]. The CT images, structures, and dose distributions were then manually rotated out of the traditional transverse, sagittal, and coronal planes to align the patient’s heart contour along the heart long-axes (LAX) and short-axes (SAX) views. MIM software was then used to sub-segment the rotated heart contour into 12 segments, relative to the heart apex, mid, and base regions (4 segments for each region), as shown in Figure 1a. Basal doses included the atria, and septal (right) doses included the right ventricle. A custom MIM workflow was created to extract dose–volume histogram (DVH) data: V5, V30, mean dose, maximum dose, and volume for all cardiac substructures and segments.

### 2.3. MRI Examination

The MRI examinations were conducted on a 3T GE MRI scanner (GE Healthcare, Milwaukee, WI, USA), which included cine sequences for measuring cardiac function, tagging for measuring regional myocardial strain, T1 and T2 sequences for tissue characterization, and perfusion and late gadolinium enhancement (LGE) sequences for detecting ischemia and previous infarction scars. For cine and LGE, a parallel stack of short-axis images covering the heart from base to apex was acquired, as well as 3 LAX slices (2-, 3-, and 4-chamber views). For tagging, T1/T2/ECV mapping, and perfusion, three SAX slices at the basal, mid-ventricular, and apical levels were acquired. Optimized MRI parameters of the different sequences used in this study are listed in Table 1, which were originally optimized by the authors to achieve maximum scan efficiency.

The cine images were analyzed using the cvi42 software version 5.16 (Circle, Calgary, AB, Canada) function module to measure the global markers, EF, and myocardial mass. The T1 and T2 images were analyzed using the cvi42 software T1 and T2 mapping modules, respectively, to generate T1, T2, and ECV maps. The SinMod method (InTag, Leon, France) [24] was used to analyze the tagged images to measure segmental myocardial strains (12 segments as shown in Figure 1), regional strain (base, mid, apex), and global strain (global longitudinal (GLS), circumferential (GCS), and radial (GRS) strains). Image analysis was conducted by a cardiac MRI physicist with 20 years of experience (E.I.) and confirmed by a cardiothoracic radiologist with 13 years of experience (A.S.).

### 2.4. Statistical Analysis

Results are represented as median ± standard error. Changes in cancer patients from baseline to post-radiation therapy were quantified by calculating the differences for each variable derived from cardiac MRI. To determine if changes were statistically significant, the non-parametric Wilcoxon signed-rank test was employed, given the small sample size and the non-normal distribution of some variables. A *p*-value of less than 0.05 was considered statistically significant. In order to assess the magnitude of these changes, Cohen’s *d* values were computed for all variables. Cohen’s *d* is a measure of effect size, which quantifies the magnitude of differences. The analysis used a threshold of 0.7 to identify variables with substantial change [25]. Further analysis on the identified variables was conducted using a mixed linear model regression, which accounts for repeated measures of the data. The mixed model included fixed effects from baseline to post-RT and random intercepts for subjects to accommodate the intra-subject correlation of the repeated measures. The results from the mixed models are represented as estimated marginal means with 95% confidence intervals and *p*-values. Correlation analysis was conducted between segmental myocardium strain and corresponding V30 measurements. We used V30 as the dose parameter in our correlation analysis due to its effectiveness as shown in previous studies [5]. We used absolute value strain in correlation analysis to be consistent among all strain components, such that positive and negative correlation coefficient values represent improved and deteriorated strains, respectively, for any strain component (circumferential, longitudinal, and radial). Figures were generated using Microsoft 365 Excel and PowerPoint software.

## 3. Results

### 3.1. Patient Characteristics

Concurrent chemotherapy was administered for ten patients in the form of weekly carboplatin and paclitaxel. Eight of the patients did not continue to the second MRI scan due to death (four patients) or withdrawal from the study. The remaining four patients (two NSCLC and two esophageal cancer; three males and one female; age 63 ± 13 years old) completed both MRI examinations. Demographics in all 12 subjects are shown in Supplementary Information. Cardiovascular risk factors included hypertension, smoking, atrial fibrillation/flutter, chest angina, acute coronary syndrome, and myocardial infarction. Comorbidities were found in five patients, which included diabetes, anemia, high cholesterol level, and pulmonary disease.

### 3.2. MRI and Dose Measurements

Baseline MRI measurements in all 12 subjects are shown in Table 2. This demonstrated regional cardiac dysfunction and elevated relaxation parameters, indicative of diffuse fibrosis or edema at baseline. Although most of these patients did not continue to the follow-up timepoint, this data demonstrates the importance of baseline cardiac MRI as a sensitive tool for enhanced evaluation of heart health before RT.

MRI measurements at baseline and follow-up timepoints in the four patients who completed the study are shown in Table 3. The MRI relaxation maps were not available in one follow-up patient due to early termination of the exam, and ECV was not available in another patient due to image artifacts. LV mass (63 ± 8 vs. 58 ± 4 g/m^2^) and T1 (1256 ± 39 vs. 1454 ± 115 ms) values, represented as median ± standard error, showed the largest differences between the baseline and follow-up timepoints, although no differences were statistically significant. The decrease in ECV could be influenced by the missing data. The rest of the parameters for the whole heart showed minimal changes between the two timepoints. Almost all strain measurements (GCS, GLS, GRS) at both baseline and follow-up timepoints were below the normal threshold absolute value of 17% (i.e., GRS < 17% while GCS and GLS > −17%) [26]. The perfusion and LGE images did not reveal ischemia or scars.

The patients had a mean heart dose of 13.2 ± 4.3 Gy (median ± standard error). The volume and radiation dose parameters (V5, V30, mean and maximum) of the cardiac substructures in patients who completed both MRI scans are shown in Supplementary Information. The spatial diversity of the included patients’ intrathoracic tumors is reflected in the large variations for the dose metrics, in particular for superior structures such as the atria. Segmental radiation dose (V30) as well as different strain components are shown in Table 4 for all 12 segments of the heart.

### 3.3. Segmental Myocardial Strain Analysis

**Figure 2:**
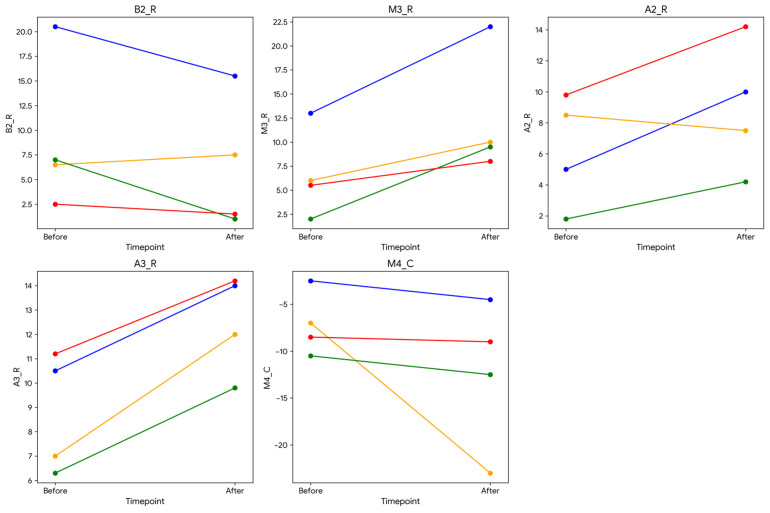
Strain parameters for segments with a large change (high Cohen’s *d* value). The segments are numbered based on the 12-segment model (B, M, A = base, mid, apical, respectively) as shown in Figure 1. The last letter represents strain (R, C = radial, circumferential, respectively). Increases and decreases in radial and circumferential strains, respectively, imply higher myocardial contractility.

None of the segmental MRI strain variables showed significant differences between baseline and follow-up (*p* > 0.05). Myocardial segments that showed large change (effect size) were: basal anteroseptal (B2_R), mid-ventricular inferoseptal (M3_R), apical anteroseptal (A2_R), and apical inferoseptal (A3_R) radial strains, and mid-ventricular inferolateral (M4_C) circumferential strain. Changes in strain in these five segments between baseline and follow-up are shown in Figure 2. The identified variables with substantial changes between baseline and follow-up were further examined using a mixed linear regression model (Table 5). The model estimated the marginal means for each variable, providing insight into the changes from baseline to follow-up (post-RT), while controlling for individual variability. The results in Table 5 indicate that the findings have probably major clinical implications.

### 3.4. Correlation Results Between MRI and Dose Parameters

**Figure 3:**
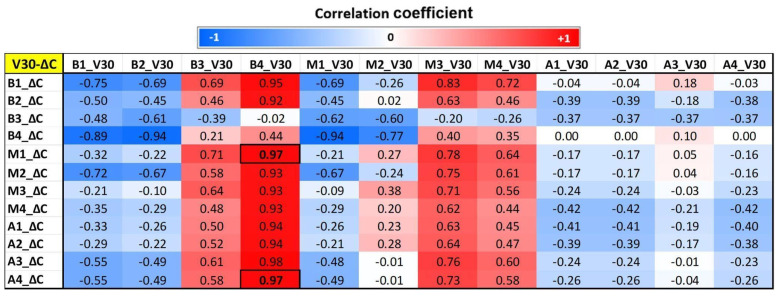
Correlation map between segmental circumferential myocardial strain and corresponding segmental V30 (V30-ΔC) in the 12 segments of the heart, as shown in Figure 1. B = base, M = mid, A = apical, ΔC = change in circumferential strain. Correlation values range from −1 (blue) to 1 (red). Significant correlations (*p* < 0.05) are shown in boldface inside boxes.

**Figure 4:**
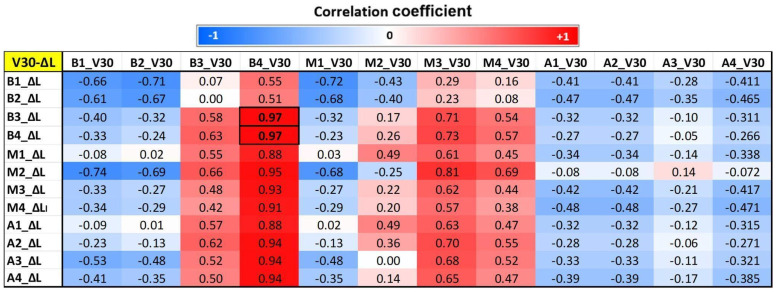
Correlation map between segmental longitudinal myocardial strain and corresponding segmental V30 (V30-ΔL) in the 12 segments of the heart, as shown in Figure 1. B = base, M = mid, A = apical, ΔL = change in longitudinal strain. Correlation values range from −1 (blue) to 1 (red). Significant correlations (*p* < 0.05) are shown in boldface inside boxes.

**Figure 5:**
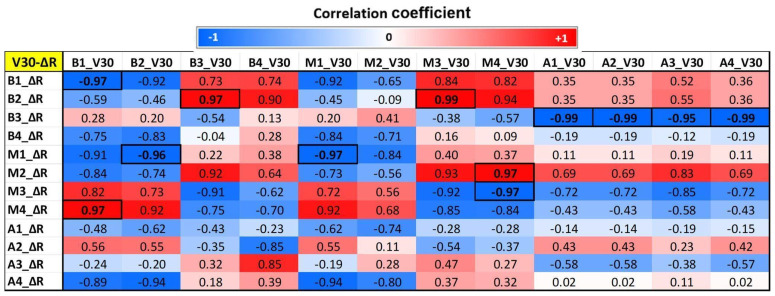
Correlation map between segmental radial myocardial strain and corresponding segmental V30 (V30-ΔR) in the 12 segments of the heart. B = base, M = mid, A = apical, ΔR = change in radial strain. Correlation values range from −1 (blue) to 1 (red). Significant correlations (*p* < 0.05) are shown in boldface inside boxes.

Figure 3, Figure 4 and Figure 5 show segmental correlation maps between V30 versus changes (follow-up—baseline) in circumferential (ΔC), longitudinal (ΔL), and radial (ΔR) strains, respectively. The correlation maps showed similar patterns (correlation values represented by red and blue colors) between dose (V30) distribution and segmental circumferential and longitudinal strains (Figure 3 and Figure 4), while the pattern was different for radial strain (Figure 5). The correlation maps showed that basal and mid-ventricle left coronary artery regions (segments B1, B2, and M1; please refer to Figure 1b) have negative mild-to-moderate correlations with circumferential and longitudinal strains. However, the basal right coronary artery territory (B3 and B4) has mild-to-moderate positive correlations with circumferential and longitudinal strains. These results can be explained based on the geographical locations of the LV segments, where segments B1, B2, and M1 are located anteriorly compared to segments B3 and B4, which are located posteriorly (Figure 1b). The results showed that radiation to the basal and mid-anterior segments has adverse effects on all segments (negative correlations or deteriorated strain), while radiation to the basal posterior segments causes remodeling (positive correlations or improved strain) of almost all LV segments. Significant correlations (*p* < 0.05) are represented by boxed boldface values in Figure 3 and Figure 4.

Regarding radial strain, the results showed that doses in anterior basal and mid segments (B1, B2, M1, M2) have negative correlations with anterior basal and mid (B1, B2, M1, M2), posterior basal (B4), and posterior apical (A4) radial strain. Additionally, doses in M3 and M4 had negative correlations with radial stain in M3 and M4. On the contrary, however, doses in the anterior basal and mid (B1, B2, M1, M2) segments have positive correlations with posterior mid (M3 and M4) radial strains. Significant correlations (*p* < 0.05) are represented by boxed boldface values in Figure 5.

## 4. Discussion

In this single-center prospective study, we used cardiac MRI for comprehensive assessment of myocardial strain and tissue composition in patients with thoracic cancer undergoing RT at baseline and six-month follow-up and correlated the results with regional heart doses. Key findings from this study are that segmental strain parameters showed potential to be sensitive markers of regional changes in cardiac function post-RT. Although statistical significance was not obtained, five of the segmental strain parameters showed notable changes between baseline and six-month follow-up (Figure 2).

On the segmental level, the results suggest that anterior basal and mid segments (M1, M2, B1, B2) may be more affected by V30 dose (high correlation coefficients) compared to the rest of the myocardial segments. This leads us to hypothesize that basal and mid territories of the left coronary artery (both left anterior descending (LAD) and left circumflex (LCX)) are more affected by radiation compared to posterior segments in the territory of the right coronary artery (RCA), where coronary artery territories are shown in Figure 1b. These results not only agree with previously reported results in the literature [11,12,13,14] but also provide more detailed information about the strain distributions of the heart on the segmental level.

The patients in this study did not show myocardial perfusion defects or fibrosis/infarction; however, it has been previously reported that RT could lead to these findings [27], which may result in arrhythmias [28]. The timepoint for this study (six months post-RT) may be too early for some of these side effects to be manifested. The results from this study also demonstrated the importance of baseline cardiac MRI examination that reveals information about cardiac function and tissue characteristics [19], which is more comprehensive and accurate than echocardiography [17]. It was noticed that almost all patients had abnormal MRI values at baseline, which is crucial for the interpretation of follow-up measurements, as previously demonstrated. Furthermore, this information can be used for patient stratification and optimized treatment management, where patients with lower myocardial strains could start a cardioprotective therapy or even treatment planning can be adjusted to avoid high radiation to segments with low strains.

Our study has some limitations. First, there is a small sample size. The number of patients who did not continue to the follow-up timepoint also highlights the challenges of studies such as these in patients with later-stage lung and esophageal cancers. While this affects the statistical significance of the results, the recorded trends and significant correlation coefficient cells in the correlation maps are promising and warrant more investigation in a larger study to confirm these results. We also suggest the inclusion of patients with lymphoma, which would add value to the study due to better prognosis in this population. Another limitation is that we focused on V30 as a measure of radiation dose due to its association with outcomes in prior studies [5]. However, low-dose (V5, V15) and high-dose (V45, V50) volume parameters have also been used in the literature [6,13,29]. Finally, although we calculated strain in the left ventricle myocardium, the dose calculation was conducted on the whole heart; therefore, doses in the anterior and septal (right) regions include the atria and right ventricle, respectively. Although the MRI strain analysis was used in this study, the addition of other important wall motion parameters, such as tissue velocity and displacement mapping, would allow for a comprehensive evaluation of heart mechanics in cancer patients undergoing RT, which is one of the aims of our future research. Finally, while we used a 12-segment model of the heart in this study due to software analysis limitations, extrapolating the results to the full AHA 16-segment model is recommended in future studies.

## 5. Conclusions

MRI provides a non-invasive means for a comprehensive assessment of the effect of RT on heart function and myocardium tissue composition in thoracic cancer patients. The association between segmental heart doses and corresponding myocardial strain analysis would allow for determining relative radiation sensitivity of different heart regions, which could lead to improvement in precision treatment, i.e., modifying treatment planning in the future to minimize radiation dose to the sensitive myocardial regions would result in reduced RT-induced cardiotoxicity and improved outcomes. Based on the promising results, a larger follow-up study is warranted.

## Figures and Tables

**Table 1:** Pulse sequence optimized imaging parameters.

Sequence	Cine	Tagging	T1 Map	T2 Map	Perfusion	LGE
Pulse seq	FIESTA	GRE	MOLLI	MESE	GRE	IR-GRE
TR (ms)	3.6	5.7	2.9	895	2.5	5.1
TE (ms)	1.3	3.1	1.3	11–77	1.7	2.3
Flip angle (°)	55	8	35	90	20	25
VPS/ETL	14	14	14	16	1	14
Averages	1	1	1	1	0.75	1
Matrix	256 × 256	256 × 256	256 × 256	256 × 256	256 × 256	256 × 256
Slice thick (mm)	8	8	8	8	8	8
BW (Hz/pixel)	488	391	977	651	326	139

Abbreviations: TR = repetition time, TE = echo time, VPS = views per segment, ETL = echo train length, BW = bandwidth, LGE = late gadolinium enhancement, FIESTA = fast imaging employing steady-state acquisition, GRE = gradient echo, MOLLI = modified Look-Locker inversion recovery, MESE = multi-echo spin echo, IR = inversion recovery.

**Table 2:** Baseline cardiac MRI parameters in all patients.

Parameter	Values in Cohort	Normal Values
LVEF (%)	57 ± 3 [50, 61]	≥50%
LV mass (g/m^2^)	63 ± 3 [49, 68]	43–115
GCS (%) *	−10.0 ± 0.5 [−11.2, −9.6]	≤−17%
GLS (%) *	−14.2 ± 0.6 [−14.7, −12.7]	≤−17%
GRS (%) *	9.6 ± 2.4 [9.2, 12.5]	≥17%
T1 (ms) *	1269 ± 28 [1251, 1376]	<1250
T2 (ms) *	52 ± 1 [47, 55]	<50
ECV (%) *	37 ± 2 [30, 43]	≤25%

Abnormal values are marked with an asterisk (*). LV = left ventricle; LVEF = left ventricular ejection fraction; GCS, GLS, GRS = global circumferential, longitudinal, and radial strains, respectively; ECV = extracellular volume. Values are represented as median ± standard error and [first quartile, third quartile].

**Table 3:** Baseline and follow-up cardiac MRI parameters.

Parameter	Baseline	Follow-Up	*p*
LVEF (%)	58 ± 3 [54, 62]	58 ± 6 [49, 66]	0.810
LV mass (g/m^2^)	63 ± 8 [53, 71]	58 ± 4 [55, 61]	0.574
GCS (%)	−10.1 ± 1.1 [−11.3, −8.8]	−12.0 ± 3.4 [−15.1, −9.3]	0.516
GLS (%)	−14.3 ± 0.7 [−14.8, −13.4]	−14.4 ± 3.1 [−17.3, −13.8]	0.513
GRS (%)	9.2 ± 2.9 [8.7, 12.2]	9.8 ± 1.0 [8.1, 11.3]	0.515
T1 (ms)	1256 ± 39 [1238, 1304]	1454 ± 115 [1333, 1563]	0.237
T2 (ms)	54 ± 5 [51, 58].	55 ± 3 [52, 57]	0.814
ECV (%)	42 ± 4 [36, 45]	33 ± 3 [31, 36]	0.533

LV = left ventricle; LVEF = left ventricular ejection fraction; GCS, GLS, GRS = global circumferential, longitudinal, and radial strains, respectively; ECV = extracellular volume. Values are represented as median ± standard error and [first quartile, third quartile].

**Table 4:** V30 and changes (Δ) in myocardial strains in the 12 cardiac segments shown in Figure 1.

Segm#	Segment Location	V30 (%)	ΔC (%)	ΔL (%)	ΔR (%)
B1	Base, Left Anterior	5.4 ± 14.7	−0.75 ± 5.64	3.00 ± 3.32	−5.00 ± 5.91
B2	Base, Right Anterior	0.2 ± 9.2	0.00 ± 2.68	4.50 ± 4.01	−3.00 ± 1.65
B3	Base, Right Posterior	0.2 ± 0.1	−1.75 ± 3.29	−3.50 ± 3.54	2.75 ± 2.52
B4	Base, Left Posterior	0.5 ± 4.1	2.25 ± 3.54	−1.75 ± 3.97	−5.75 ± 9.52
M1	Mid, Superior	0.0 ± 0.0	1.25 ± 3.99	4.00 ± 4.48	0.00 ± 5.91
M2	Mid, Anterior	0.1 ± 0.1	1.75 ± 1.99	2.25 ± 6.37	−1.75 ± 1.88
M3	Mid, Inferior	22.9 ± 15.0	0.50 ± 2.25	−0.25 ± 4.23	5.75 ± 1.51
M4	Mid, Posterior	8.0 ± 4.9	2.00 ± 3.64	0.25 ± 2.62	−1.00 ± 1.36
A1	Apex, Left Superior	0.0 ± 0.4	1.63 ± 3.85	−0.50 ± 3.90	−0.88 ± 2.60
A2	Apex, Right Superior	0.0 ± 5.1	0.13 ± 3.32	−1.88 ± 3.83	3.50 ± 1.36
A3	Apex, Right Inferior	9.9 ± 20.6	−0.50 ± 5.11	−1.38 ± 3.88	3.50 ± 0.43
A4	Apex, Left Inferior	0.2 ± 11.2	1.00 ± 5.66	−1.00 ± 3.72	1.38 ± 6.85

Values are represented as median ± standard error. ΔC, ΔL, and ΔR represent differences in the absolute value (follow-up—baseline) of circumferential, longitudinal, and radial strains, respectively.

**Table 5:** R. Mixed linear regression model parameters.

Parameter	Cohen’s *d*	Mixed Linear Model Mean (SE) [95% CI]
B2_R	0.832	2.75 (1.65) [−0.488, 5.988]
M3_R	−1.908	−5.75 (1.51) [−8.704, −2.796]
A2_R	−1.010	−2.75 (1.36) [−5.419, −0.081]
A3_R	−4.330	−3.75 (0.43) [−4.599, 2.901]
M4_C	0.704	5.13 (3.64) [−2.014, 12.264]

## Data Availability

The data are available from the corresponding author upon a reasonable request due to ongoing plans for enrolling more subjects.

## Supplementary Materials

The following supporting information can be downloaded at: https://www.mdpi.com/article/10.3390/tomography12050070/s1, Supplementary Table S1. Patient and treatment demographics; Supplementary Table S2. Radiation dose delivered to the cardiac substructures.

## Abbreviations

The following abbreviations are used in this manuscript:

RT	Radiation Therapy
LV	Left Ventricle
EF	Ejection Fraction
MRI	Magnetic Resonance Imaging
ECV	Extracellular Volume
NSCLC	Non-Small Cell Lung Cancer
CT	Computed Tomography
LAX	Long-Axis
SAX	Short-Axis
DVH	Dose–Volume Histogram
LGE	Late Gadolinium Enhancement
GLS	Global Longitudinal Strain
GCS	Global Circumferential Strain
GRS	Global Radial Strain
LAD	Left Anterior Descending
LCX	Left Circumflex
RCS	Right Coronary Artery
