# Supplementary material for: Association Between Regional Cardiac Radiation Dose and Magnetic Resonance Imaging Myocardial Contractility Parameters: A Prospective Pilot Study
Source: Tomography. 2026 May 12;12(5):70. doi: 10.3390/tomography12050070 (PMC13210886; doi:10.3390/tomography12050070)
Supplement: Supplementary file 1 [file tomography-12-00070-s001.zip › tomography-4183315-supplementary.pdf]

Supplementary Information

Association Between Regional Cardiac Radiation Dose and Magnetic Resonance Imaging Myocardial Contractility Parameters: A Prospective Pilot Study

Authors: *El-Sayed Ibrahim,* Slade Klawikowski, Lindsay Puckett, Elizabeth Gore, Dayeong An, Jakub Bychowski, Antonio Sosa, Gerard Walls, Carmen Bergom*

*Corresponding Authors *Email*: sayed.phd@gmail.com (*ORCID*: 0000-0003-4292-4669)

Supplementary Table S1. Patient and treatment demographics

Parameter	Value
Number of patients (male/female)	12 (9/3)
Age (years) - (median ± SE)	65.5±2.1
Body mass index (kg/m^2) - (median ± SE)	28.3±1.6
Patients with cardiovascular risk factor (n)	7
Patients with comorbidities (n)	5
Patients with cardiovascular disease (n)	4
Smoker (n)	7
Alcohol use (n)	5
Patients receiving beta blockers and calcium channel (n)	3
Patients receiving concurrent chemotherapy (n)	10
Patients who completed the 6-months MRI scan (n)	4

Supplementary Table S2. Radiation dose delivered to the cardiac substructures

Structure	V5 (%)	V30 (%)	Mean Dose (Gy)	Max Dose (Gy)	Volume (cc)
Heart	57±21 [28, 85]	12±4 [7, 17]	13.2±4.3 [9.9, 18.6]	50±4 [48, 55]	992±121 [812, 1110]
Left Ventricle	50±27 [6, 95]	3±5 [0, 9]	9.4±4.9 [1.5, 17.6]	29±12 [9, 47]	295±43 [234, 342]
Right Ventricle	45±28 [0, 92]	2±5 [0, 8]	10.1±4.7 [2.5, 17.5]	31±11 [13, 46]	162±23 [132, 184]
Left Atrium	74±20 [50, 89]	32±14 [17, 47]	21.2±6.9 [13.7, 27.5]	49±7 [42, 54]	90±16 [82, 103]
Right Atrium	61±25 [22, 94]	8±4 [5, 12]	13.3±5.0 [5.7, 19.9]	49±12 [36, 51]	100±25 [64, 135]
Aorta	55±3 [50, 60]	39±9 [25, 51]	21.6±3.1 [17.6, 25.0]	57±4 [51, 63]	217±109 [191, 322]
Pulmonary Artery	72±22 [51, 81]	20±15 [0, 43]	17.1±8.0 [7.5, 27.9]	46±14 [26, 60]	91±12 [79, 99]
Pericardium	57±18 [36, 77]	16±3 [13, 17]	15.0±3.1 [11.2, 17.2]	58±5 [51, 64]	1178±173 [983, 1338]

Values are represented as median ± standard error [first quartile, third quartile]
